# Power Through or Keep Looking? Comparing Species‐Area Relationships of Habitat Fragments and Their Drivers in Different Ecoregions

**DOI:** 10.1002/ece3.71928

**Published:** 2025-08-11

**Authors:** Travis S. Steffens, Alexandria E. Cosby, Mamy Razafitsalama, Shawn M. Lehman, Jean‐Luc Raharison, Mitchell T. Irwin

**Affiliations:** ^1^ Department of Sociology & Anthropology University of Guelph Guelph Ontario Canada; ^2^ Planet Madagascar Guelph Ontario Canada; ^3^ Department of Integrative Biology University of Guelph Guelph Ontario Canada; ^4^ Planet Madagascar Association Antananarivo Madagascar; ^5^ Department of Anthropology University of Toronto Toronto Ontario Canada; ^6^ SADABE Madagascar Antananarivo Madagascar; ^7^ Department of Biological Sciences Northern Illinois University DeKalb Illinois USA

**Keywords:** biogeography, diversity, lemurs, Madagascar, species richness, tropics

## Abstract

Our study aimed to (1) determine how the shape varies and mechanisms influence species‐area relationships within the same taxon but between different ecoregions and (2) determine how slope (*z*) and intercept (*c*) values of the linearized power model were influenced by ecoregion. Location: Madagascar. Taxon: Arboreal mammals (lemurs). We surveyed arboreal mammals (lemurs) in 42 tropical dry deciduous forest fragments in Ambanjabe Field Site in Ankarafantsika National Park in Western Madagascar and 27 primary mid‐elevation raiforest fragments in the Tsinjoarivo‐Ambalaomby new protected area in Eastern Madagascar using line‐transect methods. We determined which of 20 species‐area models were the most likely using the ‘*sars*’ R package and AICc in each ecoregion. We compared *z* and *c* values of the power model in each ecoregion using ANCOVA. We assessed what drove the shape of the SARs using the Measurement of Biodiversity framework. We found that SAR models differed between ecoregions, with the power model (AICc = 89.04) as the most likely in the west and the Monod model (AICc = 86.98) followed by three candidate models (Kobayashi, AICc = 87.02; logarithmic, AICc = 87.6; and negative exponential, AICc = 88.61) in the east. We found no significant difference in *z* values between ecoregions (*F*_1,66_ = 2.991, *p* = 0.088) and a non‐significant trend in *c* values between ecoregions (*F*_1,65_ = 3.938, *p* = 0.051). Spatial aggregation of species drove species richness patterns in the west, and species diversity and evenness drove species richness patterns in the east. Our study demonstrates that while the power and negative exponential model are good starting points, other models are also likely models to describe SARs in arboreal mammals such as primates. These patterns can reflect different mechanisms driving SARs. Ecoregion was not strongly related to differences in either *z* or *c* values of the power model.

## Graphical abstract

Investigating the patterns and processes that determine species‐area relations (SARs) among similar taxa that live in different ecoregions can help resolve how underlying ecologies can affect SAR patterns. Our study found that patterns of SARs varied between regions (A.) and these patterns were driven by spatial aggregation of species in western dry forests and higher species diversity and evenness in eastern rainforests (B.).

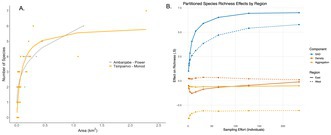

## 1 Introduction

The species‐area relationship (SAR) is one of the oldest known ecological relationships (Triantis et al. 2012). In its simplest form, SARs describe how the number of species tends to increase with area. This trend has been observed for centuries and has been explored mathematically since the 1850s (Fattorini et al. 2017; Li et al. 2020; Lomolino 2001; Rosenzweig 1995; Watson 1859; Williams 1964). The species‐area relationship is a core component of many ecological and evolutionary theories, such as the Island Biogeography Theory (MacArthur and Wilson 1967). There are at least 23 SAR models described in the literature (Dengler 2009) and most provide a simplistic framework for understanding patterns of species richness at multiple scales (Matthews, Triantis, et al. 2019). SARs have been applied to contiguous areas of increasing size (Dengler 2009), nested areas (Storch 2016), true islands (Fattorini et al. 2017; Matthews et al. 2016; Triantis et al. 2012), discrete portions of habitat in fragmented landscapes (Matthews et al. 2016; Matthews, Rigal, et al. 2019; Rabelo et al. 2017; Steffens and Lehman 2019) countries, and continents (Harcourt and Doherty 2005). While the Habitat Amount Hypothesis posits that total habitat amount is the primary determinant of species richness (Fahrig 2013), recent evidence shows that fragmentation effects, such as patch isolation and edge configurations, significantly influence SAR slopes, particularly over time (Haddad et al. 2017). Researchers have even attempted to determine what the global pattern of the SAR is and what the associated mechanisms are (Matthews, Rigal, et al. 2019). Understanding what form SARs take, what influences the shape of SARs, and how SARs differ between regions and taxonomic groups (Dengler 2009; Matthews, Rigal, et al. 2019; Scheiner 2003; Tjørve 2009) can aid in efforts to understand what influences local and historical species diversity and assembly (Caley and Schluter 1997).

### 1.1 Shape of SARs

There are two main SAR patterns, sigmoidal and convex, with numerous models that can be applied to reflect the shape of each model. Which model best reflects actual SARs has been debated since the 1920s when Arrhenius (1921) argued that SARs formed a power relationship versus the exponential model (logarithmic in non‐linear form) favored by Gleason (1922). Between the 1920s and until the 2000s the power model was dogmatically used to represent the SAR in most taxa (Scheiner 2003; Tjørve 2009). However, some researchers studying plants continued to use the exponential model (Barnett and Stohlgren 2003) likely due to its simplicity and effectiveness in describing the rapid accumulation of plant species in smaller areas (Connor and McCoy 1979). As species area research has seen a resurgence in the 21st century, scholars have applied numerous models of varying complexity to investigate the SAR in a variety of taxa under different conditions such as habitat fragmentation, environmental gradients, biogeographic regions, and disturbance regimes (Fattorini et al. 2017; Matthews et al. 2016; Passy et al. 2023). Although there is no consensus, the power model has been found to be the best model among those selected for habitat fragments and true islands (Matthews et al. 2016) including in primates (Steffens and Lehman 2019). However, the power model is not always the most likely model. For example, Guilhaumon et al. (2008) investigated how different SAR models estimated species richness in biodiversity hotspots and found that the power model was the most appropriate model in only a few situations such as in tropical grasslands for birds. Additionally, SAR shape can depend on anthropogenic factors influencing local species richness. For example, Tittensor et al. (2007) found that the magnitude of fish harvesting directly related to a similar magnitude decrease in slope in power and exponential SARs of coral reef fish regardless of reef type or taxa. Thus, unless there is a priori knowledge of the shape of the SAR for a taxa within a region and how other variables such as anthropogenic pressures affect SAR shape, one should not assume that the SAR forms a power model (Tjørve 2003; Tjørve 2009).

Scale plays an important role in SARs and can affect which model best reflects a particular SAR (Scheiner et al. 2000). Models can be applied at virtually any scale and can be used to understand scaling mechanisms in ecology. For example, the small island effect (i.e., the area under the curve where no species occur until a minimum threshold of habitat amount is met) may occur at small scales where there are species with minimum area requirements inhabiting true islands or habitat fragments (Lomolino 2000). A sigmoidal model would be the most ecologically valid in these situations if the scale range was large enough for a sigmoidal curve to form. Triantis et al. (2012) suggested that this scale range may need to be greater than three orders of magnitude. Additionally, a sigmoidal model would not be expected in a contiguous area because there would be no minimal area required for species occurrence. Evidence also suggests that SARs may not follow sigmoidal models for taxa in which some species have low minimum habitat requirements or high dispersal ability (Steffens and Lehman 2019).

The form of the SAR matters (Matthews et al. 2016) and each can represent potential hypotheses. For example, the power model represents a classic SAR (MacArthur and Wilson 1967); models such as the asymptotic, Chapman‐Richards, Gompertz, Heleg, logistic, Monod, negative exponential, and Weibull reflect SARs where there is a rapid increase in species (because of initial sampling effects; Connor and McCoy 1979) but an upper limit or a saturation point because of habitat homogeneity (Rosenzweig 1995; Scheiner 2003; Triantis et al. 2003; Williams 1964) competition (He and Legendre 2002), or reaching carrying capacity (Lawson and Jensen 2006). Models such as the Beta‐P, Extended power, Koboyashi, Persistence, and power reflect SARs influenced by more complex or multiple factors (Matthews et al. 2016).

### 1.2 Power Model and the Meaning of *c* and *z* Values

The power model was favored through the 20th century because of its perceived wide applicability, relative simplicity (i.e., 2 parameters—*c* and *z*), and ease in linearizing the model ($\text{Species RichnessRichness}=c\times A^{z}$ becomes logSpecies RichnessRichness=logc+logA×z; Matthews et al. 2016). In this linear form, c and z are fitted constants representing the intercept and slope. Canonical versions of these parameters (especially z) have been of interest to SAR researchers for decades (Lomolino 2000; Preston 1962). How c and z vary between SARs could provide information about the processes that lead to the formation of SARs in the first place. *C* values have been observed to increase when diversity increases (Patiño et al. 2014) and are lower in oceanic versus continental islands (Matthews et al. 2016). Z values vary among taxa, between taxa, at multiple scales, and in different regions (Matthews et al. 2016; Tjørve and Tjørve 2008; Triantis et al. 2012). Researchers have suggested that *z* tends to be higher in continents versus continental islands versus oceanic islands (Patiño et al. 2014), and that *z* values will increase with area, isolation, trophic rank, nestedness, spatial aggregation of individuals, human impact, and latitude (see Fattorini et al. 2017 for review). However, Fattorini et al. (2017) found homogeneous *z* values but significant differences in *c* values in SARs among the same taxon within different island groups that varied in their isolation and paleogeographical history. Therefore, more research is needed to determine how *c* and *z* values vary at different spatial scales.

### 1.3 Primates

Primates have relatively high levels of species richness for tropical mammals, and primate researchers have historically been interested in SARs within the taxon (Harcourt and Doherty 2005; Lehman 2004; Marshall et al. 2010). Primate researchers have investigated how the SAR describes primate species richness at local (Steffens and Lehman 2019), regional (Lehman 2004; Marshall et al. 2010), and global scales (Harcourt and Doherty 2005). Although there has been some research on the shape of primate SARs and the influence of variables other than area on primate species richness (e.g., Steffens and Lehman 2019), no research has been conducted to compare SARs and the value of *c* and *z* in the power model between ecoregions within this taxon. Steffens and Lehman (2019) found that area explained the most variance in species richness in Ankarafantsika National Park in Northwestern Madagascar, even when considering other habitat characteristics and anthropogenic factors. They also found that lemurs form a convex rather than a sigmoidal SAR. They suggest that this SAR forms a convex shape because of the relative ubiquity of mouse lemurs in even the smallest fragments (e.g., 0.0023 km^2^) and the capacity for those species to disperse among fragments (Steffens et al. 2022; Steffens and Lehman 2018). Similarly, Setash et al. (2017) highlight that mouse lemur density across Madagascar is influenced by the interplay between habitat fragmentation, seasonal resource availability, and species' adaptations to heterogeneous environments, which may explain the observed convex patterns.

Lemurs in Madagascar provide an opportunity to determine what is the most likely SAR shape and how c and z parameters of the power model vary within the same taxon but between different ecoregions. Lemurs are widely distributed and there is an east/west gradient in elevation and rain with higher and more varied elevation and wet forests in the east, and lower, flatter elevation and dry forests in the west (Jury 2022). Lemur species richness tends to be greater in the eastern wet forests (Ganzhorn 1997; Steffens et al. 2022); conversely, species' densities tend to be higher (i.e., smaller home ranges) in western forests (Ganzhorn et al. 2006; Hending 2021; Setash et al. 2017). For example, Hending (2021) suggests variation in *Cheirogaleus* density related to elevation, temperature, and precipitation and may reflect east–west variations similar to those observed in *Microcebus* (Setash et al. 2017). There is an east/west gradient in mouse lemur home range size (Radespiel 2006). For example, eastern *M. rufus* have reported home ranges from 0.3 to 0.7 ha (Dammhahn and Kappeler 2005) compared western *M. murinus* have ranges which have reported home ranges from 0.7 (Eberle and Kappeler 2004) – 3.2 ha (Pages‐Feuillade 1988). In eastern wet forests *Propithecus diadema* have reported home ranges between 10.9–90.2 ha (Irwin and Raharison 2021) while *P. coquereli* and *P. verreauxi* in western dry forests have reported home ranges ranging between 4.4 and 19.81 ha (McGoogan 2011; Richard 1985) and 5.7–10.1 ha (Benadi et al. 2008) resp**e**ctively with one study reporting home ranges as low as 1–8 ha for both species (Richard 1985).

The purpose of our study was to determine how the shape of SAR (i.e., reflecting underlying processes creating SARs) and how the values of *c* and *z* parameters of the linearized power model varied within the same taxon but between different ecoregions. We tested two hypotheses: (1) Lemur SAR models in each region are best described by a convex upward model as found by Steffens and Lehman (2019) and (2) the *c* and *z* parameters will not and will vary respectively, between regions. However, we expect that there will be a difference in which convex models are the best‐fit models in each ecoregion. Because of higher population densities and lack of small island effects the SAR in the west will be represented by a power model (Steffens and Lehman 2019). Because of lower population densities, the SAR in the east will be represented by a more complex SAR model (Irwin and Raharison 2021; Setash et al. 2017) Comparing log–log power models we predict c values to be similar between the east versus west because the similar overall species richness observed in each study site (Tsinjoarivo) has relatively lower species richness than other eastern sites.

We expect that the western dry deciduous forests will have higher z‐values than humid eastern rainforests because of the greater density of lemurs in the west, making them more resilient to reductions in fragment size (Hending 2021; Setash et al. 2017). Higher densities in the west can be attributed to the more dietary plastic community assemblage, which exhibits greater dietary and ecological flexibility necessitated by seasonally unpredictable resources, enabling species to persist in fragmented and resource‐limited habitats (Ganzhorn et al. 1999; Ossi and Kamilar 2006; Sato et al. 2014). This resilience contrasts with the eastern region, where species rely on the more consistent availability of high‐quality but patchily distributed resources, making them less tolerant of fragmentation (Ganzhorn et al. 1999; Setash et al. 2017). Additionally, the uniform anthropogenic grasslands in the west act as a homogeneous matrix that limits dispersal (Steffens and Lehman 2018), thereby concentrating populations within fragments and amplifying species turnover as fragment size decreases. In contrast, the eastern forests feature a more heterogeneous matrix, including secondary forests and agroforestry landscapes, which facilitates higher dispersal and connectivity between fragments, reducing the steepness of the species‐area relationship (Ndriantsoa et al. 2017). The combination of higher densities and restricted dispersal in the west supports greater species richness in smaller fragments, contributing to higher *z*‐values, while the connectivity afforded by the heterogeneous eastern matrix buffers against species loss, resulting in lower *z*‐values.

## 2 Methods

### 2.1 Study Site

#### 2.1.1 Ambanjabe (West)

**FIGURE 1:**
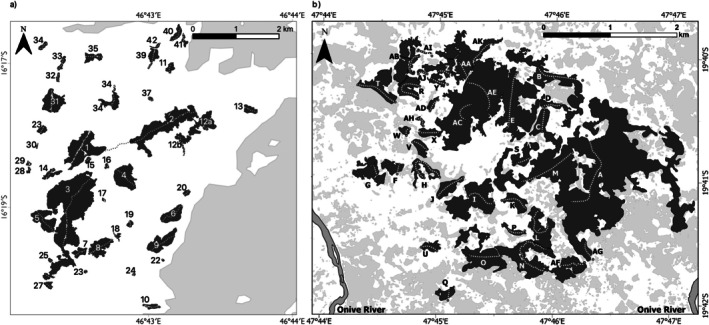
(a) Ambanjabe field site location in Ankarafantsika National Park in the dry deciduous forest of western Madagascar. (b) Mahatsinjo field site located in the Tsinjoarivo rainforest in eastern Madagascar.

Ambanjabe is an 80 km^2^ conservation management zone within Ankarafantsika National Park (Figure 1a). Ankarafantsika National Park is 1300 km^2^ in size and located in western Madagascar. The park consists primarily of dry deciduous forest that is mostly continuous but fragmented along the periphery of the park. The park contains some of the last remaining relatively intact portions of dry deciduous forest in Madagascar (Schüßler et al. 2023; Waeber et al. 2015). Ambanjabe is on the western edge of Ankarafantsika National Park and is characterized by a series of forest fragments ranging in size from 0.0023 km^2^ to 1.177 km^2^, surrounded by anthropogenic grassland and bounded by continuous forest to the north, east, and south. Although the anthropogenic grassland matrix between the fragments is mostly homogeneous, there are drainage lines, and isolated bushes and trees that may act as linear corridors and stepping‐stones for species to disperse among fragments (Steffens and Lehman 2021). On the eastern edge of Ambanjabe are three communities along a river valley that runs approximately north–south. Within Ambanjabe there are eight species of lemurs, including six that have been recorded within the fragments (Table 1).

**TABLE 1:** Lemur species within Ankarafantsika National Park.

Lemur species	Activity pattern	Diet	IUCN Red List status
*Avahi occidentalis*	Nocturnal	Folivore	Vulnerable
*Cheirogaleus medius* ^a^	Nocturnal	Omnivore	Vulnerable
*Eulemur fulvus* ^a^	Cathemeral	Frugivore	Vulnerable
*Eulemur mongoz*	Cathemeral	Frugivore	Critically endangered
*Lepilemur edwardsi* ^a^	Nocturnal	Folivore	Endangered
*Microcebus murinus* ^a^	Nocturnal	Omnivore	Least concern
*Microcebus ravelobensis* ^a^	Nocturnal	Omnivore	Vulnerable
*Propithecus coquereli* ^a^	Diurnal	Folivore‐frugivore	Critically endangered

^a^ Observed in this study.

#### 2.1.2 Tsinjoarivo (East)

Tsinjoarivo forest is in the western sector of the Tsinjoarivo‐Ambalaomby new protected area (256.87 km^2^; Figure 1b). The study area, Mahatsinjo, covers roughly 20 km^2^ on the western edge of the protected area (and therefore the highest elevations, at the transition between the largely denuded central plateau grasslands and the eastern rainforests), and is heavily deforested and fragmented due to population influxes from Madagascar's central plateau. The vegetation is primarily mid‐elevation rainforest (forest covers roughly half of the Mahatsinjo region), and ranges in elevation from roughly 1500–1650 m. The twenty‐seven forest fragments studied ranged from 0.0046 to 2.28 km^2^. Average rainfall is 2100 mm per year, of which 63% falls in the rainy season from December through March (Irwin, unpub. data). Due to its high altitude, Mahatsinjo's temperatures are low for Malagasy rainforests: daily average maxima range from 17.2 to 26.4 C (July and December respectively) and daily average minima range from 6.6 to 14.4 C (July and January respectively). There are 10 lemur species recorded in Tsinjoarivo (Table 2).

**TABLE 2:** Lemur within Tsinjoarivo.

Lemur species	Activity pattern	Diet	IUCN Red List status
*Avahi laniger* ^a^	Nocturnal	Folivore	Vulnerable
*Cheirogaleus crossleyi* ^a^, ^b^	Nocturnal	Frugivore	Vulnerable
*Cheirogaleus sibreei* ^a^, ^b^	Nocturnal	Frugivore	Critically endangered
*Daubentonia madagascariensis* ^a^	Nocturnal	Omnivore	Endangered
*Eulemur fulvus*	Cathemeral	Frugivore	Vulnerable
*Eulemur rubriventer*	Cathemeral	Frugivore	Vulnerable
*Hapalemur griseus* ^a^	Nocturnal	Folivore	Vulnerable
*Lepilemur mustenlinus* ^a^	Nocturnal	Folivore	Endangered
*Microcebus lehilahytsara* ^a^	Nocturnal	Frugivore‐Insectivore	Near threatened
*Propithecus diadema* ^a^	Nocturnal	Folivore‐frugivore	Critically endangered

^a^ Observed in this study.

^b^ Combined as single species.

### 2.2 Data Collection

#### 2.2.1 Ambanjabe

We conducted visual surveys along line transects in 42 fragments between June 14 and November 20 in 2011 by (Steffens and Lehman 2019). One line transect was established along the longest path through each fragment while still going through the center of the fragment (Figure 1). Four transects (R2, 18, 19, and 37) also extended into the matrix. Each line transect was visually surveyed between 11 and 21 times in the day and 11 and 18 times in the night to facilitate observation of nocturnal, diurnal, and cathemeral lemur species. One species in Ambanjabe, *C. medius* , hibernates between April and October (Dausmann et al. 2005). However, this species became active and was very conspicuous in Ambanjabe between October and November, so field teams ensured that all fragments were surveyed again during this period. Although there were different survey efforts in each transect, all transect species accumulation curves reached an asymptote over the course of the surveys. Because of the difficulty surveying arboreal primates, for analysis purposes, we treat the entire season as a single snapshot of the species occurrences within each fragment. For a more detailed explanation of the methods, see Steffens and Lehman (2018, 2019) and Steffens, Mercado Malabet, and Lehman (2020).

#### 2.2.2 Tsinjoarivo

We conducted visual surveys along line transects between September 7th and October 29th, 2001. Between one and four line transects were placed within 27 forest fragments (Figure 1b). Each transect was visually surveyed between eight to ten times at night and 17–18 times during the day to facilitate observation of nocturnal, diurnal, and cathemeral lemur species until all species accumulation curves reached asymptotes. Two nocturnal species, *C. crossleyi* and *C. sibreei* (identified to the genus level in this study), hibernate until at least September (Blanco and Rahalinarivo 2010; McLain et al. 2017) and like in Ambanjabe, these *Cheirogaleus* spp. became active in mid‐October during this study (first sighted on 15 October). We surveyed each transect nocturnally four to five times following the first *Cheirogaleus* observation.

### 2.3 Analysis

To test if ecoregion affected the SAR shape we used the ‘*sars*’ R package to fit 20 non‐linear SAR models for each dataset and compared them using AICc to determine which was the best‐fit model (Matthews, Triantis, et al. 2019). We considered models as candidate models if they were within 2 AICc values of the model with the lowest AICc (Burnham and Anderson 2002). We also determined how much variation in species richness was explained by area by comparing Adjusted‐*R* ^2^ values for each model. We then applied the model averaging feature in the ‘*sars*’ R package on only candidate SAR models to determine what was the shape of the SAR for each data set.

To test how c (intercept) and z (slope) of the Power model differed between regions we compared a linearized log–log (using a log_10_) version of the model (log *S =* log *cc + z* log × *A*) where S = species richness and A = area using an ANCOVA. In the ANCOVA we compared the mean values for log species richness (dependent variable) with corresponding log area values (covariate) with region as the factor. We then conducted an *F*‐test to compare models where region was added to the model as only an interactive factor and then as an interactive and an additive factor. If the interaction between the factor and area was significant then we determined the z values (slopes) were different between regions. If the additive factor (region) and area were both found to be significant then we determined that there was a significant difference in c (intercept) values between regions. We also assessed the performance of the linearized Power model using the ‘*sars*’ R package (Matthews, Triantis, et al. 2019). We tested for homoscedasticity in each model by comparing a correlation of the residuals and the model fitted values (Quinn and Keough 2002). We checked for normality of the residuals in each model by applying the Lilliefors extension of the Kolmogorov–Smirnov normality test (Quinn and Keough 2002).

To create species accumulation curves (rarefaction curves) we used the *iNex'* R package (3.01; Hsieh et al. 2024; Chao et al. 2014), with cumulative individual‐based abundance and species richness data. To test if species richness significantly increased with area rather than sampling effort, we compared the rarefied species richness at standardized coverage against fragment area for both regions using ‘*iNext*’. We assessed what was driving regional lemur SAR patterns across fragmented landscapes using the Measurement of Biodiversity framework (MoB; McGlinn et al. 2019) and the ‘*mob*’ R package (3.02; McGlinn et al. 2024) to implement scale‐explicit partitioning of species richness. We compared the observed species richness and abundance of each fragment (including fragment area) within and between each region (i.e., east and west) to assess the effect of three mechanisms on species richness: (1) the effect of individual density, (2) spatial aggregation, and (3) species abundance distribution. Sampling effort was standardized using individual‐based rarefaction curves.

## 3 Results

**FIGURE 2:**
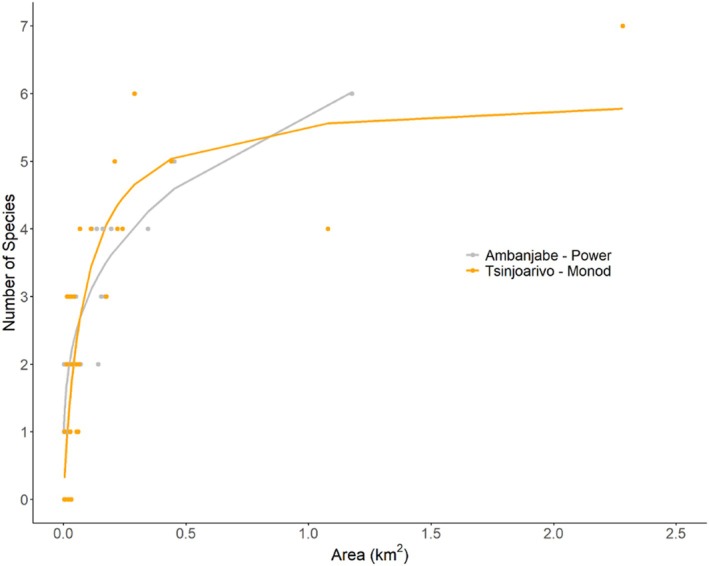
Species area relationship model for the best‐fit models of lemur species richness for the Ambanjabe field site (Power) in western Madagascar, and the Tsinjoarivo field site (Monod) in eastern Madagascar.

We were able to fit 20 models to each dataset. For both regions, we found that the best fit and all candidate species area models were convex upward models (Tables 1 and 2; Figures S1 and S2). At Ambanjabe, we found that the Power model had the lowest AICc value and was the best‐fit model among the 20 models (Figure 2; Table 3). There were no other candidate models (i.e., models within 2 AICc of the most likely model). In Ambanjabe, we found that the Power model log area explained 71.3% of the variation in log species richness (Figure 2; Table 3). In Tsinjoarivo, we found that the Monod model was the best‐fit model, followed by three other candidate models (Kobayashi, Kobayashi, logarithmic, and negative exponential; Table 4). In the Tsinjoarivo SAR models, log area explained less variation (between 62.727% and 64.949%; Table 4) than the SAR models in Ambanjabe (Table 3). While models within 4–7 AIC values are considered valuable (Burnham and Anderson 2002; Richards 2005), more complex models like Chapman‐Richards and Heleg may add unnecessary complexity (Reineking and der 2006). We recommend a stricter delta AICc ≤ 2 threshold to avoid overfitting and maintain biologically meaningful interpretations, especially in studies with small sample sizes.

**TABLE 3:** Species‐area model comparison for 20 models in Ambanjabe Field Site.

Model	Weight	AICc	Adjusted *R* ^2^	Shape	Asymptote
**Power**	**0.264**	**89.039**	**0.713**	**Convex/up**	**FALSE**
Extended Power 2	0.092	91.160	0.708	Sigmoid	FALSE
Persistence Function 2	0.089	91.222	0.707	Sigmoid	FALSE
Power Rosenzweig	0.082	91.385	0.706	Convex/up	FALSE
Extended power 1	0.081	91.412	0.706	Convex/up	FALSE
Heleg (Logistic)	0.078	91.489	0.706	Convex/up	FALSE
Weibull‐3	0.078	91.489	0.706	Convex/up	FALSE
Persistence Function 1	0.078	91.489	0.706	Convex/up	FALSE
Chapman‐Richards	0.078	91.489	0.706	Convex/up	FALSE
Weibull‐4	0.021	94.075	0.698	Convex/up	FALSE
Asymptotic	0.019	94.301	0.685	Convex/up	TRUE
Beta‐P	0.018	94.361	0.696	Convex/up	FALSE
Kobayashi	0.014	94.909	0.670	Convex/up	FALSE
Logarithmic	0.005	96.979	0.653	Convex/up	FALSE
Logistic	0.003	97.693	0.659	Sigmoid	TRUE
Rational	0.001	100.986	0.631	Convex/up	FALSE
Monod	0.000	109.047	0.538	Convex/up	TRUE
Linear	0.000	111.318	0.512	Linear	FALSE
Negative Exponential	0.000	118.471	0.422	Convex/up	TRUE
Gompertz	0.000	122.629	0.382	Convex/down	FALSE

*Note:* Bold represents candidate models (i.e., within 2 AICc values of the lowest model). Adjusted *R* ^2^ values are calculated by the ‘*sars*’ R package in (Matthews, Triantis, et al. 2019).

**TABLE 4:** Species‐area model comparison for 20 models in Tsinjoarivo.

Model	Weight	AICc	Adjusted *R* ^2^	Shape	Asymptote
**Monod**	**0.165**	**86.975**	**0.649**	**Convex/up**	**TRUE**
**Kobayashi**	**0.162**	**87.019**	**0.649**	**Convex/up**	**FALSE**
**Logarithmic**	**0.120**	**87.608**	**0.641**	**Convex/up**	**FALSE**
**Negative Exponential**	**0.073**	**88.611**	**0.627**	**Convex/up**	**TRUE**
Heleg (Logistic)	0.054	89.212	0.641	Convex/up	TRUE
Chapman‐Richards	0.051	89.336	0.640	Convex/up	TRUE
Weibull‐3	0.051	89.345	0.640	Convex/up	TRUE
Asymptotic	0.048	89.430	0.638	Convex/up	TRUE
Extended power 1	0.048	89.449	0.638	Convex/up	FALSE
Gompertz	0.038	89.896	0.632	Sigmoid	TRUE
Power Rosenzweig	0.034	90.109	0.629	Convex/up	FALSE
Logistic	0.029	90.438	0.625	Sigmoid	TRUE
Persistence Function 2	0.029	90.445	0.625	Sigmoid	FALSE
Power	0.029	90.461	0.601	Convex/up	FALSE
Extended Power 2	0.024	90.825	0.619	Sigmoid	FALSE
Persistence Function 1	0.020	91.193	0.614	Convex/up	FALSE
Beta‐P	0.012	92.247	0.625	Convex/up	TRUE
Weibull‐4	0.002	92.344	0.624	Convex/up	TRUE
Rational	0.000	99.330	0.478	Convex/up	FALSE
Linear	0.000	104.241	0.335	Linear	FALSE

*Note:* Bold represents candidate models (i.e., within 2 AICc values of the lowest model). Adjusted *R* ^2^ values are calculated by the R ‘*sars*’ package in Matthews, Rigal, et al. (2019), Matthews, Triantis, et al. (2019).

**FIGURE 3:**
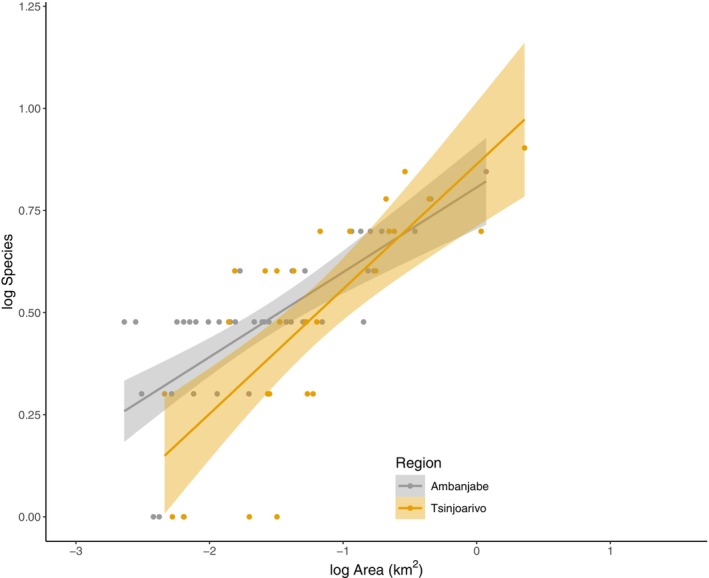
Linear regressions of log_10_‐transformed lemur species richness (log_10_ Species) against log_10_ ‐transformed fragment area (log_10_ Area) for Ambanjabe Field Site (Adjusted $R^{2}=0.538,F_{1,40}=48.83,P<0.01,n=42$) and Tsinjoarivo (Adjusted $R^{2}=0.550,F_{1,25}=32.71,P<0.01,\;n=27$). Ambanjabe: Log*S* = log (1.858 ± 0.117) + log*A* (0.208 ± 0.030). Tsinjoarivo: Log*S* = log (1.988 ± 0.17) + log*A* (0.306 ± 0.053). Errors refer to standard error.

Based on a plot of SAR regression slopes and standard error values for each region there are visual differences in the intercept and slope (Figure 3). However, based on the ANCOVA results (Table 5) we found that there was a trend towards significance between the c values (intercept) (*F*_1,65_ = 3.938, *p* = 0.051) between regions but we did not find significant differences in the z values (slope) between regions (*F*_1,66_ = 2.991, *p* = 0.088).

**TABLE 5:** ANCOVA results comparing log–log power model *z* and *c* values between Ambanjabe Field Site and Tsinjoarivo.

	Sum of squares	df	*F*	*p*
**Model 1: log (species+1) = log (area) + Zone + log (area):Zone**				
Log (area)	9.354	1	79.585	< 0.01
Zone	0.477	1	4.057	0.048
Log (area):Zone	0.352	1	2.992	0.088
**Model 2: log (species+1) = log (area) + Zone**				
Log (area)	9.354	1	77.253	< 0.01
Zone	0.477	1	3.938	0.051

**FIGURE 4:**
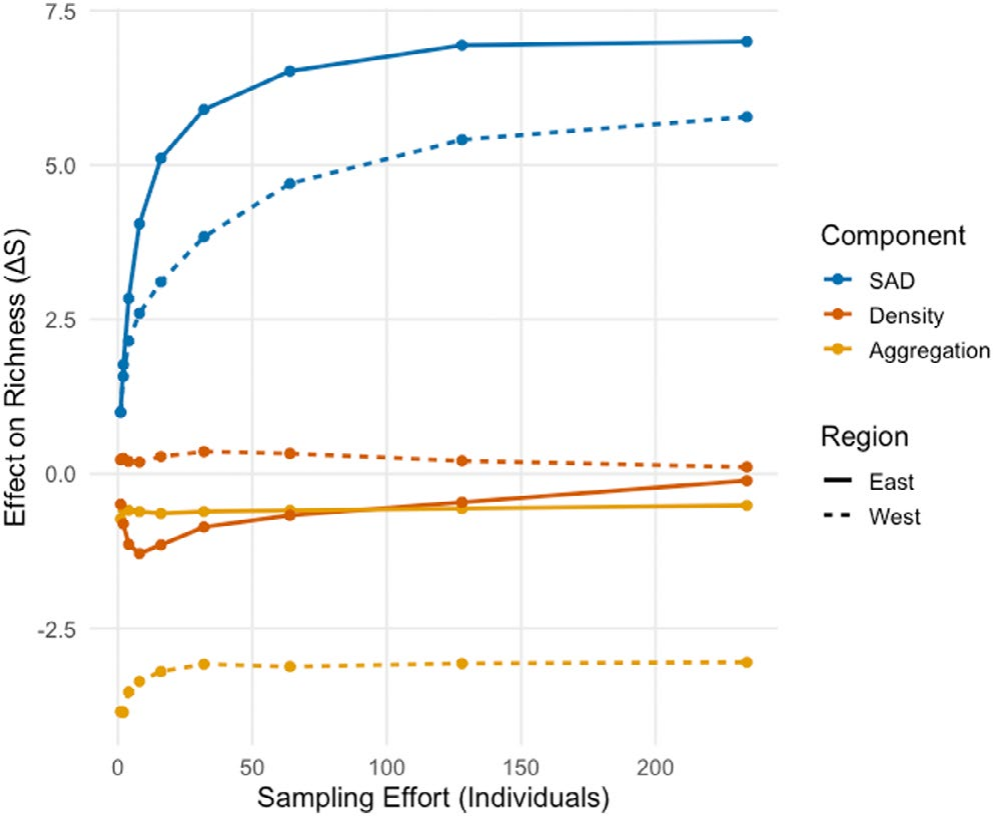
Effect of species abundance distribution (SAD), density, and spatial aggregation on species richness in Ambanjabe Field Site (west) and Tsinjoarivo (east).

When accounting for sampling effort, we found fragment area was a significant predictor of species richness in both the west (*β* = 3.88 ± 0.65 SE, *p* < 0.001) (*R* ^2^ = 0.50) and east (*β* = 2.37 ± 0.63 SE, *p* = 0.001), explaining approximately 48.9% and 37.2% of the variance (Adjusted *R* ^2^) respectively (Table 6). Using the MoB framework, we confirmed that regional species richness patterns were not because of sampling effort, as richness differences persist even when effort is equalized across regions (Figure 4; Table S1). We found that density had a weak and non‐significant effect on richness in the west, and it was not a driver of species richness in the east or explained overall differences between regions. There was a large negative effect of spatial aggregation in the west (i.e., species were more clumped in larger fragments) but not in the east, where species were more evenly distributed (Figure 4; Table S1). The eastern region is richer because it has greater species diversity and more evenly distributed species (Figure 4; Table S1).

**TABLE 6:** Regression results comparing rarefied species richness at standardized coverage against fragment area in Ambanjabe Field Site and Tsinjoarivo.

	Estimate	SE	*t*	*p*
**Ambanjabe Field Site**				
(Intercept)	2.1153	0.1447	14.624	< 0.01
Area	3.8822	0.6472	5.998	< 0.01
**Tsinjoarivo**				
(Intercept)	9.354	1	77.253	< 0.01
Area	0.477	1	3.938	0.051

## 4 Discussion

Fitting SAR shapes helps to determine the mechanisms that drive the SAR pattern (Matthews et al. 2016; Tjørve 2009; Triantis et al. 2012). Ambanjabe and Tsinjoarivo SARs were both convex, despite being composed of a matrix of habitable patches (forest fragments) and uninhabitable patches such as grassland and agricultural areas. We did not detect a small‐island effect in either region as the fragment size difference between the smallest and largest fragments was less than four orders of magnitude (Triantis et al. 2012). Studies have shown that lemurs, especially *Microcebus* spp., can survive in small fragments (Steffens and Lehman 2019), have high dispersal capacity between fragments (Steffens and Lehman 2018), can survive in fragments and matrix elements for extended periods of time (Irwin et al. 2010; Kling et al. 2020; Steffens and Lehman 2021), and have been observed surviving in degraded patches and agroecosystems, including vanilla plantations and other agricultural landscapes, where they can use these habitats as extensions of natural forests or as travel corridors (Knoop et al. 2018; Hending et al. 2018; Webber et al. 2019). Although dispersal is difficult to measure directly in primates, lemurs have been observed to cross gaps between forest fragments (Eppley et al. 2015; Ganzhorn and Schmid 1998) or use matrix habitat to facilitate movement (Steffens and Lehman 2021). For example, Eppley et al. (2015) found that the southern bamboo lemur ( *Hapalemur meridionalis* ) would move into and use matrix habitat as dispersal corridors. Steffens, Ramsay, et al. (2020) found that *Microcebus* spp. would occupy matrix elements along linear paths between fragments – using these as corridors to move through relatively hostile matrix. Similar findings were observed for frog movement through matrix in eastern fragmented landscapes that varied in matrix quality where frogs preferentially moved through higher quality matrix elements (Ndriantsoa et al. 2017). Further research is n**e**eded to understand how primates move through different matrix types, and how this affects their dispersal capacity in fragmented versus continuous forest.

The debate on which SAR curve fits best has persisted since the early 1920s (Arrhenius 1921; Gleason 1922). Although the power model is widely applied, it may not the best fit (Guilhaumon et al. 2008). More studies are now applying other models to SAR data (Dengler 2009; Matthews et al. 2016; Triantis et al. 2012). However, a few studies have investigated other curve fits for SARs in primates, e.g., Benchimol and Peres (2013) and Steffens and Lehman (2019). Our study found the power model best fit in Ambanjabe but the Monod model was best fit Tsinjoarivo followed by the Kobayashi, logarithmic, and negative exponential models. These models different families, logarithmic (Kobayashi, and logarithmic), logistic (Monod), and negative exponential.

Selecting a better fit model to represent a SAR is important because even subtle variations among models can have important implications in predicting species richness at extreme areas (small or large). For example, in the Tsinjoarivo dataset, it would require a substantial increase in habitat size to increase one species in the logarithmic versus Kobayashi (both among the exponential family; Triantis et al. 2012) versus power model, which is typically applied to SAR datasets. Our MoB analysis detected differences in the shape of the SAR in each region. This analysis demonstrated that density did not have effects on species richness in the west, but spatial aggregation of species did. Higher species diversity and evenness were driving the shape of the SAR in the east. These results have direct conservation implications. For example, in the west, the change in species richness is driven by clumping at large fragment sizes, meaning that losing large fragments will have a greater effect than the absolute loss of similar area among smaller fragments. In the east, small changes in fragment size at all, including the smaller fragment sizes (~10 to 80 ha) will result in greater reductions of species richness than in the west.

Understanding the importance of c (intercept) and z (slope) values has been debated among SAR researchers (Tjørve and Tjørve 2008), but few studies have investigated because there are too many variables that contribute to differences c and z values between taxa and study sites (Brown and Lomolino 1998; Connor and McCoy 1979). Comparing the linear version of the power model (log species richness ~ log area) in the same taxa in different regions provides a useful opportunity to understand what impacts c and z values (Fattorini et al. 2017; Gould 1979). In Ambanjabe the matrix is mostly homogeneous anthropogenic grassland whereas in Tsinjoarivo the matrix is more heterogeneous. We expected this difference to allow greater dispersal, and thus lower z values, in Tsinjoarivo, but found no significant difference in z values between sites. This may be because the matrix in Ambanjabe is not necessarily more hostile or that some species are able to occupy and move through the matrix (Steffens and Lehman 2021). Additionally, there are two mouse lemur species in Ambanjabe and one in Tsinjoarivo which could offset the effect of matrix hostility. We expected a difference in c values (i.e., intercept) when species richness is higher (Fattorini et al. 2017). Controlling for z we found that c was trending higher in the more species rich eastern region (Tsinjoarivo) than in the less species rich western region (Ambanjabe). Lemurs in western Madagascar are denser but less species‐rich than in the east (Ganzhorn et al. 2006; Setash et al. 2017), which may be due to greater habitat heterogeneity in the east, such as altitudinal gradients and increased tree diversity (Lehman 2014; Ganzhorn et al. 1997). Notably the power model was not a good fit for Tsinjoarivo and therefore, the differences or lack thereof between *z* and *c* values between regions may reflect the fact that the power model was simply not a good fit in the east.

## 5 Conclusion

Primates are a useful group to test hypotheses about the cause and shape of SARs. They have relatively high species richness, occur in highly fragmented habitats, and are complex social species. By comparing SARs between biogeographic ecoregions we cannot conclude that the power model is the most appropriate model for all scenarios. However, if the power model is a candidate model comparing c and z values between similar taxa in different habitats provides an opportunity to understand how habitat type and possibly local fragmentation effects impact SAR shape. By using the MoB framework, we demonstrate that the SARs in each region are driven by factors other than sampling effects such as density, spatial aggregation, and evenness. Our study has important implications for conservation, particularly in the context of ongoing threats to species habitats. Understanding how different SAR models apply to fragmented landscapes and what mechanisms are driving these patterns can inform strategies for managing and conserving biodiversity under increasing threats.

## Author Contributions

**Travis S. Steffens:** conceptualization (lead), data curation (lead), formal analysis (lead), funding acquisition (lead), investigation (lead), methodology (lead), project administration (lead), visualization (lead), writing – original draft (lead), writing – review and editing (lead). **Alexandria E. Cosby:** formal analysis (supporting), writing – original draft (supporting), writing – review and editing (supporting). **Mamy Razafitsalama:** investigation (supporting), project administration (supporting), writing – review and editing (supporting). **Shawn M. Lehman:** conceptualization (supporting), funding acquisition (supporting), methodology (supporting), supervision (lead), writing – original draft (supporting), writing – review and editing (supporting). **Jean‐Luc Raharison:** investigation (supporting), methodology (supporting), project administration (supporting), writing – review and editing (supporting). **Mitchell T. Irwin:** conceptualization (equal), data curation (equal), formal analysis (supporting), funding acquisition (equal), investigation (equal), methodology (equal), project administration (equal), writing – original draft (supporting), writing – review and editing (supporting).

## Ethics Statement

The authors have nothing to report.

## Consent

All co‐authors have consented to the publication of this manuscript.

## Conflicts of Interest

The authors declare no conflicts of interest.

## Supporting information


**Appendix S1:** ece371928‐sup‐0001‐AppendixS1.docx.

## Data Availability

The data used for this research, including the species rarefaction curves and raw data, can be found in Appendix S1, Figures 3 and 4 and Tables 2 and 3, respectively.
