# Supplementary material for: Power Through or Keep Looking? Comparing Species‐Area Relationships of Habitat Fragments and Their Drivers in Different Ecoregions
Source: Ecol Evol. 2025 Aug 11;15(8):e71928. doi: 10.1002/ece3.71928 (PMC12339419; doi:10.1002/ece3.71928)
Supplement: Supplementary file 1 — Appendix S1: ece371928‐sup‐0001‐AppendixS1.docx. [file ECE3-15-e71928-s001.docx]

**Supplementary Information**  **
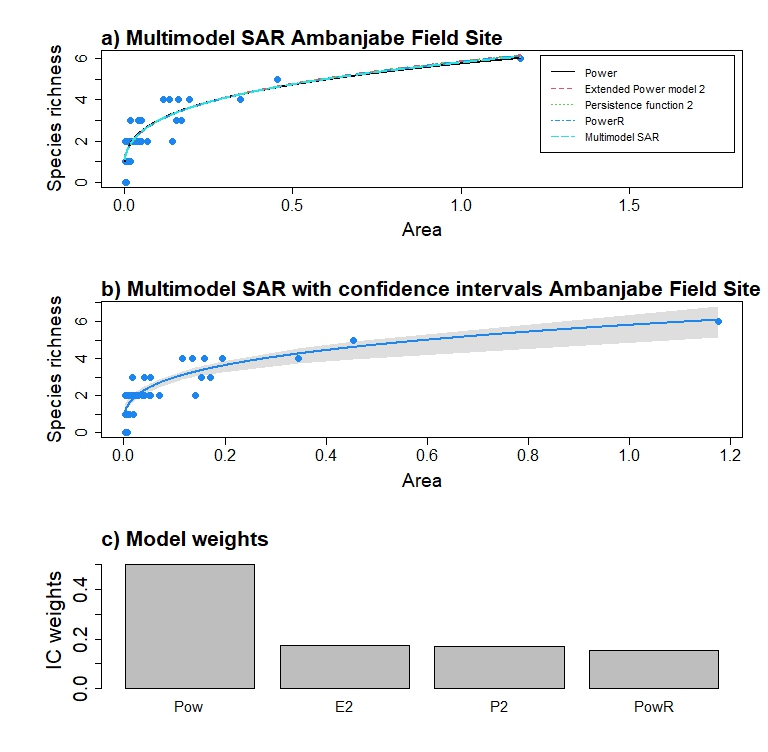
**

SI Figure 1: Species Area Relationship models displaying the best-fitted model (power) for the Ambanjabe field site in western Madagascar.


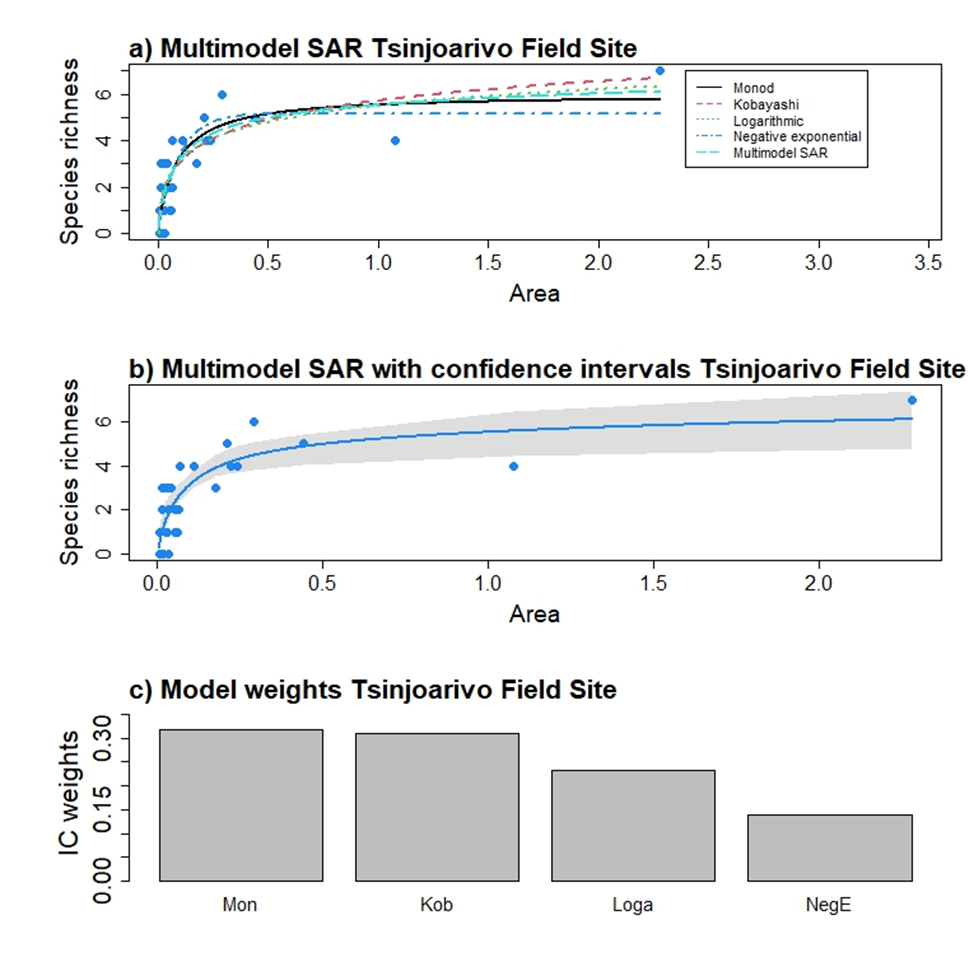


SI Figure 2: Species Area Relationship models displaying the best-fitted model (Monod) for the Tsinjoarivo field site in eastern Madagascar.

SI Table 1: Effect of Species Abundance Distribution (SAD), density of species, and spatial aggregation of species while accounting for sampling effort on species richness between Ambanjabe (west) and Tsinjoarivo (east).

|  | Group | Test | Sample | Effort | S | Effect | 95% Confidence Interval |
| --- | --- | --- | --- | --- | --- | --- | --- |
| 1 | west | SAD | indiv | 1 | 1.00 | 1 | 1-1 |
| 2 | west | SAD | indiv | 2 | 1.58 | 1.58 | 1.7-1.73 |
| 3 | west | SAD | indiv | 4 | 2.15 | 2.15 | 2.59-2.72 |
| 4 | west | SAD | indiv | 8 | 2.60 | 2.6 | 3.6-3.89 |
| 5 | west | SAD | indiv | 16 | 3.11 | 3.11 | 4.88-5.37 |
| 6 | west | SAD | indiv | 32 | 3.84 | 3.84 | 6.64-7.3 |
| 7 | west | SAD | indiv | 64 | 4.70 | 4.7 | 8.51-9.42 |
| 8 | west | SAD | indiv | 128 | 5.41 | 5.41 | 10.1-11.2 |
| 9 | west | SAD | indiv | 234 | 5.78 | 5.78 | 10.9-12.2 |
| 10 | west | N | indiv | 1 | 1.23 | 0.23 | -0.286-0.257 |
| 11 | west | N | indiv | 2 | 1.83 | 0.253 | -0.491-0.423 |
| 12 | west | N | indiv | 4 | 2.35 | 0.198 | -0.743-0.593 |
| 13 | west | N | indiv | 8 | 2.79 | 0.191 | -0.918-0.66 |
| 14 | west | N | indiv | 16 | 3.39 | 0.282 | -0.883-0.562 |
| 15 | west | N | indiv | 32 | 4.20 | 0.359 | -0.719-0.478 |
| 16 | west | N | indiv | 64 | 5.03 | 0.33 | -0.687-0.44 |
| 17 | west | N | indiv | 128 | 5.61 | 0.206 | -0.683-0.431 |
| 18 | west | N | indiv | 234 | 5.89 | 0.109 | -0.695-0.317 |
| 19 | west | agg | plot | 1 | 2.31 | -1.2 | -3.85--3.45 |
| 20 | west | agg | plot | 2 | 2.95 | -1.42 | -3.86--3.27 |
| 21 | west | agg | plot | 3 | 3.26 | -1.59 | -3.53--2.8 |
| 22 | west | agg | plot | 4 | 3.55 | -1.62 | -3.36--2.47 |
| 23 | west | agg | plot | 5 | 3.79 | -1.58 | -3.2--2.28 |
| 24 | west | agg | plot | 6 | 4.12 | -1.39 | -3.08--2.1 |
| 25 | west | agg | plot | 7 | 4.55 | -1.06 | -3.12--2.09 |
| 26 | west | agg | plot | 8 | 4.71 | -0.974 | -3.07--2.05 |
| 27 | west | agg | plot | 9 | 4.90 | -0.844 | -3.05--2.01 |
| 28 | west | agg | plot | 10 | 5.19 | -0.605 | -2.47--1.56 |
| 29 | west | agg | plot | 11 | 5.31 | -0.524 | -2.38--1.4 |
| 30 | west | agg | plot | 12 | 5.36 | -0.508 | -2.17--1.21 |
| 31 | west | agg | plot | 13 | 5.40 | -0.486 | -2.24--1.28 |
| 32 | west | agg | plot | 14 | 5.43 | -0.484 | -2.2--1.21 |
| 33 | west | agg | plot | 15 | 5.50 | -0.43 | -2.12--1.15 |
| 34 | west | agg | plot | 16 | 5.57 | -0.372 | -2.05--1.12 |
| 35 | west | agg | plot | 17 | 5.62 | -0.337 | -1.97--1.04 |
| 36 | west | agg | plot | 18 | 5.62 | -0.346 | -1.93--1.03 |
| 37 | west | agg | plot | 19 | 5.62 | -0.354 | -1.86--0.998 |
| 38 | west | agg | plot | 20 | 5.64 | -0.337 | -1.86--1.02 |
| 39 | west | agg | plot | 21 | 5.69 | -0.294 | -1.65--0.849 |
| 40 | west | agg | plot | 22 | 5.71 | -0.274 | -1.49--0.66 |
| 41 | west | agg | plot | 23 | 5.71 | -0.277 | -1.42--0.566 |
| 42 | west | agg | plot | 24 | 5.74 | -0.256 | -1.28--0.327 |
| 43 | west | agg | plot | 25 | 5.79 | -0.21 | -1.21--0.305 |
| 44 | west | agg | plot | 26 | 5.86 | -0.14 | -1.18--0.213 |
| 45 | west | agg | plot | 27 | 5.95 | -0.0456 | -0.977--0.0432 |
| 46 | east | SAD | indiv | 1 | 1.00 | 1 | 1-1 |
| 47 | east | SAD | indiv | 2 | 1.77 | 1.77 | 1.68-1.75 |
| 48 | east | SAD | indiv | 4 | 2.84 | 2.84 | 2.51-2.78 |
| 49 | east | SAD | indiv | 8 | 4.05 | 4.05 | 3.43-4.04 |
| 50 | east | SAD | indiv | 16 | 5.11 | 5.11 | 4.55-5.63 |
| 51 | east | SAD | indiv | 32 | 5.90 | 5.9 | 6.1-7.68 |
| 52 | east | SAD | indiv | 64 | 6.52 | 6.52 | 7.91-10 |
| 53 | east | SAD | indiv | 128 | 6.94 | 6.94 | 9.22-11.9 |
| 54 | east | SAD | indiv | 234 | 7.00 | 7 | 10-13 |
| 55 | east | N | indiv | 1 | 5.10 | -0.49 | -0.377-0.309 |
| 56 | east | N | indiv | 2 | 9.56 | -0.809 | -0.497-0.339 |
| 57 | east | N | indiv | 4 | 1.70 | -1.14 | -0.489-0.254 |
| 58 | east | N | indiv | 8 | 2.75 | -1.29 | -0.39-0.252 |
| 59 | east | N | indiv | 16 | 3.96 | -1.15 | -0.478-0.382 |
| 60 | east | N | indiv | 32 | 5.04 | -0.862 | -0.661-0.489 |
| 61 | east | N | indiv | 64 | 5.85 | -0.672 | -0.745-0.413 |
| 62 | east | N | indiv | 128 | 6.48 | -0.459 | -0.556-0.258 |
| 63 | east | N | indiv | 234 | 6.89 | -0.113 | -0.417-0.185 |
| 64 | east | agg | plot | 1 | 2.67 | -1.58 | -0.734--0.434 |
| 65 | east | agg | plot | 2 | 4.04 | -1.15 | -0.584-0.142 |
| 66 | east | agg | plot | 3 | 4.85 | -0.839 | -0.59-0.266 |
| 67 | east | agg | plot | 4 | 5.26 | -0.731 | -0.606-0.266 |
| 68 | east | agg | plot | 5 | 5.59 | -0.588 | -0.639-0.257 |
| 69 | east | agg | plot | 6 | 5.74 | -0.609 | -0.61-0.292 |
| 70 | east | agg | plot | 7 | 5.96 | -0.521 | -0.59-0.285 |
| 71 | east | agg | plot | 8 | 6.07 | -0.509 | -0.564-0.287 |
| 72 | east | agg | plot | 9 | 6.56 | -0.12 | -0.508-0.249 |
| 73 | east | agg | plot | 10 | 6.67 | -0.0846 | -0.483-0.247 |
| 74 | east | agg | plot | 11 | 6.78 | -0.0281 | -0.463-0.228 |
| 75 | east | agg | plot | 12 | 6.89 | 0.0333 | -0.462-0.219 |
| 76 | east | agg | plot | 13 | 6.89 | -0.00584 | -0.372-0.185 |
| 77 | east | agg | plot | 14 | 6.89 | -0.0332 | -0.396-0.236 |
| 78 | east | agg | plot | 15 | 6.96 | 0.0172 | -0.415-0.208 |
| 79 | east | agg | plot | 16 | 6.96 | -0.000543 | -0.366-0.167 |
| 80 | east | agg | plot | 17 | 7.00 | 0.0249 | -0.308-0.146 |
| 81 | east | agg | plot | 18 | 7.00 | 0.0155 | -0.291-0.154 |
| 82 | east | agg | plot | 19 | 7.00 | 0.00911 | -0.285-0.147 |
| 83 | east | agg | plot | 20 | 7.00 | 0.00535 | -0.272-0.151 |
| 84 | east | agg | plot | 21 | 7.00 | 0.00269 | -0.272-0.123 |
| 85 | east | agg | plot | 22 | 7.00 | 0.00119 | -0.223-0.112 |
| 86 | east | agg | plot | 23 | 7.00 | 0.000488 | -0.184-0.113 |
| 87 | east | agg | plot | 24 | 7.00 | 0.000135 | -0.162-0.0753 |
| 88 | east | agg | plot | 25 | 7.00 | 0.0000207 | -0.115-0.073 |
| 89 | east | agg | plot | 26 | 7 | 0.00000106 | -0.0726-0.0398 |
| 90 | east | agg | plot | 27 | 7 | 0 | 0-0 |

SI Figure 3. Rarefaction curves for species richness using abundance data for Ambanjabe (west).

SI Figure 4. Rarefaction curves for species richness using abundance data for Tsinjoarivo (east).

SI Table 2. Lemur species abundance data for Ambanjabe

| Site | Area | Unit | *Cheirogaelus medius* | *Microcebus murinus* | *Microcebus ravelobensis* | *Propithecus coquereli* | *Eulemur fulvus* | *Lepilemur edwardsi* |
| --- | --- | --- | --- | --- | --- | --- | --- | --- |
| F1 | 0.3451 | km^2^ | 1 | 17 | 28 | 0 | 1 | 0 |
| F2 | 0.4534 | km^2^ | 2 | 56 | 71 | 1 | 6 | 0 |
| F3 | 1.177 | km^2^ | 6 | 53 | 72 | 11 | 22 | 5 |
| F4 | 0.1946 | km^2^ | 0 | 18 | 19 | 6 | 4 | 0 |
| F5 | 0.1601 | km^2^ | 1 | 32 | 25 | 0 | 4 | 0 |
| F6 | 0.1158 | km^2^ | 2 | 32 | 20 | 0 | 0 | 1 |
| F7 | 0.0416 | km^2^ | 1 | 2 | 7 | 0 | 1 | 0 |
| F8 | 0.1355 | km^2^ | 0 | 18 | 11 | 0 | 2 | 0 |
| F9 | 0.1538 | km^2^ | 2 | 20 | 24 | 0 | 0 | 0 |
| F10 | 0.0278 | km^2^ | 0 | 18 | 13 | 0 | 0 | 0 |
| F11 | 0.0257 | km^2^ | 0 | 2 | 6 | 0 | 0 | 0 |
| F12.1 | 0.0375 | km^2^ | 0 | 11 | 14 | 0 | 0 | 0 |
| F12.2 | 0.0408 | km^2^ | 1 | 6 | 8 | 0 | 0 | 0 |
| F13 | 0.0118 | km^2^ | 0 | 9 | 10 | 0 | 0 | 0 |
| F14 | 0.0076 | km^2^ | 0 | 10 | 5 | 0 | 0 | 0 |
| F15 | 0.0042 | km^2^ | 0 | 11 | 13 | 0 | 0 | 0 |
| F16 | 0.0114 | km^2^ | 0 | 0 | 2 | 0 | 0 | 0 |
| F17 | 0.014 | km^2^ | 0 | 0 | 0 | 0 | 0 | 0 |
| F18 | 0.0143 | km^2^ | 0 | 2 | 2 | 0 | 0 | 0 |
| F19 | 0.0038 | km^2^ | 0 | 4 | 3 | 0 | 0 | 0 |
| F20 | 0.0023 | km^2^ | 0 | 11 | 9 | 0 | 0 | 0 |
| F21 | 0.0248 | km^2^ | 0 | 0 | 0 | 0 | 0 | 0 |
| F22 | 0.0028 | km^2^ | 0 | 2 | 2 | 0 | 0 | 0 |
| F23 | 0.0057 | km^2^ | 0 | 10 | 7 | 0 | 0 | 0 |
| F24 | 0.0197 | km^2^ | 0 | 2 | 3 | 0 | 0 | 0 |
| F25 | 0.0064 | km^2^ | 0 | 2 | 5 | 0 | 0 | 0 |
| F27 | 0.0071 | km^2^ | 0 | 3 | 1 | 0 | 0 | 0 |
| F28 | 0.0031 | km^2^ | 0 | 4 | 2 | 0 | 0 | 0 |
| F29 | 0.1703 | km^2^ | 0 | 1 | 0 | 0 | 0 | 0 |
| F30 | 0.0098 | km^2^ | 0 | 0 | 0 | 0 | 0 | 0 |
| F31 | 0.0216 | km^2^ | 3 | 6 | 12 | 0 | 0 | 0 |
| F32 | 0.0169 | km^2^ | 0 | 12 | 5 | 0 | 0 | 0 |
| F33 | 0.05 | km^2^ | 0 | 5 | 4 | 0 | 0 | 0 |
| F34 | 0.0697 | km^2^ | 2 | 14 | 19 | 0 | 0 | 0 |
| F35 | 0.0064 | km^2^ | 0 | 5 | 6 | 0 | 0 | 0 |
| F36 | 0.0052 | km^2^ | 1 | 11 | 12 | 0 | 0 | 0 |
| F37 | 0.0517 | km^2^ | 0 | 0 | 0 | 0 | 0 | 0 |
| F38 | 0.0378 | km^2^ | 0 | 1 | 1 | 0 | 0 | 0 |
| F39 | 0.0156 | km^2^ | 0 | 23 | 18 | 0 | 0 | 0 |
| F40 | 0.0079 | km^2^ | 0 | 10 | 12 | 0 | 0 | 0 |
| F41 | 0.1422 | km^2^ | 0 | 5 | 7 | 0 | 0 | 0 |
| F42 | 0.0518 | km^2^ | 0 | 2 | 11 | 0 | 0 | 0 |

SI Table 3. Lemur species abundance data for Tsinjoarivo

| Site | Area | Unit | P. diadema | Hapalemur griseus | Avahi laniger | | L. microdon | Cheirogaleus Spp. | M. rufus | D. madagascariensis |
| --- | --- | --- | --- | --- | --- | --- | --- | --- | --- | --- |
| A_C_M_AG | 2.2811 | km^2^ | 4 | 7 | | 7 | 20 | 2 | 2 | 1 |
| B_D | 0.2904 | km^2^ | 7 | 5 | | 0 | 18 | 1 | 2 | 1 |
| E_AC_AE | 1.0792 | km^2^ | 0 | 2 | | 11 | 8 | 0 | 4 | 0 |
| F | 0.0427 | km^2^ | 0 | 2 | | 1 | 0 | 0 | 1 | 0 |
| G | 0.0540 | km^2^ | 0 | 0 | | 2 | 0 | 0 | 0 | 0 |
| H_J | 0.1757 | km^2^ | 0 | 3 | | 2 | 5 | 0 | 0 | 0 |
| I | 0.2416 | km^2^ | 3 | 1 | | 1 | 2 | 0 | 0 | 0 |
| K | 0.0530 | km^2^ | 0 | 0 | | 2 | 0 | 1 | 0 | 0 |
| L_N_AF | 0.4400 | km^2^ | 8 | 3 | | 1 | 12 | 0 | 0 | 1 |
| O | 0.2094 | km^2^ | 3 | 2 | | 4 | 1 | 0 | 1 | 1 |
| P | 0.0198 | km^2^ | 0 | 0 | | 0 | 0 | 0 | 0 | 0 |
| Q | 0.0319 | km^2^ | 0 | 0 | | 5 | 1 | 0 | 0 | 0 |
| R | 0.0673 | km^2^ | 0 | 2 | | 1 | 4 | 0 | 2 | 0 |
| S | 0.0636 | km^2^ | 0 | 0 | | 1 | 2 | 0 | 0 | 0 |
| T | 0.1123 | km^2^ | 0 | 1 | | 2 | 2 | 1 | 0 | 0 |
| U | 0.0281 | km^2^ | 0 | 4 | | 0 | 0 | 0 | 0 | 0 |
| V | 0.0335 | km^2^ | 0 | 0 | | 1 | 0 | 0 | 1 | 0 |
| W | 0.0139 | km^2^ | 0 | 1 | | 0 | 0 | 0 | 2 | 0 |
| X | 0.0320 | km^2^ | 0 | 0 | | 0 | 0 | 0 | 0 | 0 |
| Y | 0.0261 | km^2^ | 0 | 3 | | 1 | 0 | 0 | 2 | 0 |
| Z | 0.0596 | km^2^ | 0 | 0 | | 1 | 0 | 0 | 0 | 0 |
| AA_AK | 0.2207 | km^2^ | 0 | 3 | | 4 | 3 | 0 | 2 | 0 |
| AB | 0.0272 | km^2^ | 0 | 1 | | 4 | 0 | 0 | 0 | 0 |
| AD | 0.0154 | km^2^ | 0 | 1 | | 0 | 9 | 0 | 1 | 0 |
| AH | 0.0064 | km^2^ | 0 | 0 | | 0 | 0 | 0 | 0 | 0 |
| AI | 0.0046 | km^2^ | 0 | 0 | | 1 | 0 | 0 | 0 | 0 |
| AJ | 0.0053 | km^2^ | 0 | 0 | | 0 | 0 | 0 | 0 | 0 |
